# Diseases of the Respiratory Organs

**Published:** 1905-06-24

**Authors:** 


### PROGRESS IN MEDICINE AND SURGERY.

### DISEASES OF THE RESPIRATORY ORGANS.

(*Continued from page* 209.)

**Tuberculin.**—Beranek's [15] tuberculin is strongly recommended by so great an authority as Trudeau, who has employed it for many years. Of his early cases in which it was not used there were at the time of writing 61 per cent. alive, whilst of similar cases in which tuberculin was given the number was 76 per cent. P. Jacobs [16] found that comparatively large quantities of fluid could be injected into the lungs by a catheter passed into the large bronchi, without causing distress or bad symptoms, and thought that a direct application of drugs in this manner was likely to be effective. He first treated five tuberculous cows with tuberculin and other drugs introduced through the trachea and found marked improvement, especially when tuberculin was used. He then administered similar infusions to human beings, and found that they were harmless and a useful mode of giving the remedy. If there is a tubercular lesion in the lung a reaction is given by a tracheal injection of tuberculin, even when the dose is only one-tenth or one-twelfth of that needed for reaction in the ordinary way. Trudeau, Baldwin, and Kinghorn [17] have carried out investigations on the action of tuberculin. They found that when there was a tuberculous lesion in the cornea of a rabbit, tuberculin exercised a healing influence. When bacilli were enclosed in a capsule and placed in the peritoneum, and tuberculin injected into the animal, no local or general reaction occurred. Hence no dialysable products of the bacillus are capable of causing a reaction, but only the bacilli themselves. Neither do they believe that the action is due to an enzyme. The reaction may take place as early as the third or fourth day after infection and is constant after the first fortnight. Bandelier [18] points out the great diagnostic value of tuberculin in early phthisis, where it is often most difficult to say whether a lesion is due to a commencing attack or to an old process which has healed already. He has never known any serious harm from a proper use of the test. The patient's temperature should have been taken every two hours for some days previously to show the absence of any fever, and the first injection should be in the daytime, lest any slight rise be overlooked. He uses 1 mg. for the first dose, 5 mg. for the second, and 10 mg. for the third, and, if no reaction follows, a fourth of 10 mg. also. Out of 500 of his sanatorium patients 34·6 per cent. reacted to 1 mg., 31 to 5 mg., 19·6 to 10 mg., and 7 per cent. to the second injection of 10 mg. There were seven per cent. who showed no reaction. These were sent home, but warned to report themselves to their own doctors. Not one of these was brought back to the sanatorium in two years.

**Serum diagnosis** has been rendered more accurate by Arloing and Courmont,[19] who have met with great difficulties in obtaining cultures of a fixed and standardised power. Each culture is measured by a human serum of known reaction. In making a test, Arloing allows a time limit of three to five hours, and dilutions as low as 1 in 20, 1 in 10, or even 1 in 5. Positive results are given by about 80 per cent. of actual tuberculous cases. The most advanced cases may fail to react, and the value of the method is greater in early stages of the disease.

*Lanoline* is said to be a remedy of great value against tuberculosis. D. McDonald [20] makes a curious assertion that the tubercle bacillus is largely composed of fat and that this is identical in composition with lanoline. When large quantities of this fat are administered the bacilli undergo a fatty degeneration and are easily destroyed by the cells of the body. The lanoline can be given by enema, by inunction or with food; patients gain weight very rapidly when taking it. He points out that sheep and goats are largely immune to tuberculous disease. *Sea voyages* for the treatment of tuberculosis have drawbacks as well as advantages. The *Lancet* [21] thinks that the tourist hospital ship sent out by the Hamburg-American line may afford to some sick persons a useful trip. Provided the diet is well planned, and a route which ensures fair weather be taken, it is possible in such a voyage to obtain fresh air and sunshine and freedom from dust. There will indeed be difficulties in providing exercise for those who require it, and of course persons who are bad sailors should not attempt such expeditions, however carefully the route be planned. Cases of advanced disease, or those of the hectic or hæmorrhagic type, or persons suffering from intestinal lesions, should not be tempted to join. Young people with a family history of phthisis, cases of suspicious pleurisy, or of tuberculous affections of the joints or glands, may indeed derive much benefit provided that they are able to live on deck and not in close cabins.

**Hæmoptysis.**—C. H. Cattle[22] refers to mistakes in diagnosis due to hæmorrhage from polypi and other nasal affections as well from laryngeal, buccal, and pharyngeal lesions. Hæmoptysis may also be due to thoracic aneurism, to bronchitis and bronchiectasis, to heart disease, and in elderly rheumatic, gouty, or emphysematous patients there may be degenerate vessels. If it comes from the lungs the presumption is that it is due to phthisis, but we often find no physical signs, hence a prolonged examination is useless. Complete rest in bed will usually put an end to the hæmorrhage if the lung disease is at an early stage. Morphine, except there is so much bleeding as to flood the air-passages, relieves both the cough and the patient's mind. Salines every hour, to produce free purgation, are all important unless the bleeding has been very severe. Calcium chloride in 20-grain doses every four hours should be given for a few days, and repeated after intervals of a week. The injection of half a pint of gelatine solution into the rectum three times a day has been warmly recommended. An ice-bag may be applied to the chest to quiet the heart. A rational plan, too, is the inhalation of nitrite of amyl, as recommended by Hare, of Brisbane; but adrenalin, by raising pressure, tends to prevent the formation of a clot, and he considers it dangerous.

[15] Scottish Med. and Surg. Jour., Feb. [16] Amer. Jour. of Med. Sci., March. [17] Jour. of Med. Research, Aug. [18] Brit. Med. Jour., Epitome, Dec. 3. [19] Boston Med. and Surg. Jour., Dec. 8. [20] Med. Rec., March 4. [21] Lancet, Jan. 14. [22] Brit. Med. Jour., Jan. 14.

